# Nicotinic Receptor Alpha7 Expression during Tooth Morphogenesis Reveals Functional Pleiotropy

**DOI:** 10.1371/journal.pone.0036467

**Published:** 2012-05-30

**Authors:** Scott W. Rogers, Lorise C. Gahring

**Affiliations:** 1 Geriatric Research, Education and Clinical Center, Veteran's Administration, Salt Lake City, Utah, United States of America; 2 Department of Neurobiology and Anatomy, University of Utah School of Medicine, Salt Lake City, Utah, United States of America; 3 Division of Geriatrics, Department of Internal Medicine, University of Utah School of Medicine, Salt Lake City, Utah, United States of America; City of Hope National Medical Center and Beckman Research Institute, United States of America

## Abstract

The expression of nicotinic acetylcholine receptor (nAChR) subtype, alpha7, was investigated in the developing teeth of mice that were modified through homologous recombination to express a bi-cistronic IRES-driven tau-enhanced green fluorescent protein (GFP); alpha7GFP) or IRES-Cre (alpha7Cre). The expression of alpha7GFP was detected first in cells of the condensing mesenchyme at embryonic (E) day E13.5 where it intensifies through E14.5. This expression ends abruptly at E15.5, but was again observed in ameloblasts of incisors at E16.5 or molar ameloblasts by E17.5–E18.5. This expression remains detectable until molar enamel deposition is completed or throughout life as in the constantly erupting mouse incisors. The expression of alpha7GFP also identifies all stages of innervation of the tooth organ. Ablation of the alpha7-cell lineage using a conditional alpha7Cre×ROSA26-LoxP(diphtheria toxin A) strategy substantially reduced the mesenchyme and this corresponded with excessive epithelium overgrowth consistent with an instructive role by these cells during ectoderm patterning. However, alpha7knock-out (KO) mice exhibited normal tooth size and shape indicating that under normal conditions alpha7 expression is dispensable to this process. The function of ameloblasts in alpha7KO mice is altered relative to controls. High resolution micro-computed tomography analysis of adult mandibular incisors revealed enamel volume of the alpha7KO was significantly reduced and the organization of enamel rods was altered relative to controls. These results demonstrate distinct and varied spatiotemporal expression of alpha7 during tooth development, and they suggest that dysfunction of this receptor would have diverse impacts upon the adult organ.

## Introduction

Nicotinic acetylcholine receptors (nAChR), in addition to their well-known contributions to neurotransmission, phenotypes of addiction, and relationship to multiple psychiatric disorders, also participate in a wide variety of physiological processes in both neuronal and non-neuronal systems throughout the body [1]–[3]. These receptors are also expressed during prenatal development, although little is known about their temporal and spatial patterning and how this could have an impact upon development and the successful completion of these processes. In this study we examined the expression of nicotinic receptor alpha7 (α7) during tooth development and innervation. The tooth is an excellent model of ectoderm organ formation, innervation and specialization. One example of the significant advantage the tooth organ offers is that its development is well characterized [4]–[7]. Teeth arise from ectoderm patterning in a series of events that begins with the thickening of oral epithelium and subsequent accumulation of cells that are recruited from the cranial neural crest that form the underlying mesenchyme [4], [8]–[10]. Also characteristic of the dramatic changes in tissue remodeling leading to the adult tooth is innervation by the trigeminal nerve [11]–[15]. Finally, cells are functionally specialized to deposit and assemble enamel and dentine prior to tooth eruption [4]–[7]. Throughout recent studies examining α7 expression during prenatal mouse developmental [16] we noted considerably spatial and temporal alterations in the expression of this receptor during different stages of ectodermal pattering, innervation and cell specialization. Our results provide evidence that α7 expression and functional pleiotropy is to be expected and it contributes to successful tooth organogenesis.

## Materials and Methods

### Animals

All animal use was in accordance with the Guide for the Care and use of Laboratory Animals of the National Institutes of Health. Animal protocols were approved in advance by the Institutional Animal Care and Use Committee at the University of Utah and assigned a Protocol Number (09-07003). Animals are housed according to NIH and IACUC guidelines.

### *Chrna7*-HA-IRES-tauGFP (α7^GFP^) and *Chrna7*-HA-IRES-Cre (α7^Cre^) mice

The development and use of reporter mice for α7 expression have been described [16]. Briefly, the α7 gene (*Chrna7*) was modified using the methods of homologous recombination. At the C-terminus of α7 an epitope extension (hemaggluttinin (HA) and stop codon) was introduced together with a bi-cistronic IRES-tauGFP reporter cassette insert [16], [17]. This generated the *Chrna7*-HA: IRES-tauGFP mouse (α7^GFP^) which expresses the tauGFP protein as a marker of *Chrna7* transcription. The resulting line was subsequently backcrossed to the C57BL/6 or C3H background for three generations using the Speed Congenic program (Jackson Laboratories Mouse Services) to achieve 98% of the desired strain congenicity. The α7-HA-IRES-Cre line was produced by replacing the tauGFP cassette with the Cre-recombinase (α7^Cre^; [16]). The ROSA26-LoxP(conditional diphtheria toxin (DTA)) mouse lines and their use in conjunction with the α7^Cre^ mouse is described elsewhere [16].

### Immunohistochemistry and Microscopy

Basic immunohistochemical methods for these studies are as before [16]. Briefly embryos from impregnated mice (coital plugs equal E0.5) were removed at indicated times and fixed in 2% paraformaldehyde, cryoprotected with sucrose and mounted in embedding medium for sectioning on a Microm EM550 microtome. Prepared sections (10 microns) in different sectioning planes (sagittal, coronal and horizontal) were made from no less than 8 different animals at each developmental stage were incubated with the appropriate combination of 1° antibody (e.g., chicken anti-GFP from AVES or others listed in text and references) overnight at 4°C. Sections are washed extensively and reacted with fluorescently labeled secondary antibodies (Jackson ImmunoResearch). C57BL/6 wild-type mice served as negative controls for immunohistochemical specificity (not shown). Examination of enamel using phase microscopy was done on 15 micron thick sections prepared from mandibular incisors. The sections (sagittal orientation) were mounted in mounting medium and photographed under fluorescence or phase optics as shown in the text and described in [16]. Antibodies included; chicken anti-GFP (1∶150; Aves), Goat anti-EDAR (1∶20; R&D Systems), rabbit anti-Cux1 (1∶100; SantaCruz), mouse anti-HA (1∶10; Sigma-Aldrich) or rabbit anti-HA (1∶20; Covance, shown); mouse anti-TuJ (1∶50; Covance), rabbit IBA1 (1∶75; Abcam). 4′,6-diamidino-2-phenylindole (DAPI) was from (Sigma-Aldrich).

### Micro-Computed Tomography (μCT) Analysis

Mice were given a lethal dose of anesthetic, their heads removed and briefly fixed in 4% paraformaldehyde before being washed in PBS. Heads were then scanned using a General Electric Medical Systems EVS-RS9 scanner (high resolution scan of 27 microns; University of Utah Small Animal Imaging Core). The resulting data were processed using General Electric Microview software (v2.1.2, GE Healthcare) to create three-dimensional reconstruction of images for subsequent analyses. Surface rendering was done using a surface quality factor of 0.9 without correction for decimation. Surface smoothing was enabled to create the images shown. For enamel volume calculations, coronal cross sections at ∼0.5 mm distance were collected for each mandibular incisor pair around the site of tooth emergence from the bone. The 2D cross-section images of the entire mandibular tooth group were matched for anatomical similarity and settings adjusted to visualize enamel as black and the remaining tooth in grey. For calculations, the cross-sectional tooth volume of each mandibular incisor was determined after coloring it to red and the enamel yellow whereupon pixel number in each color could be scored using the ImagePro Plus (MediaCybernetics) software package. The density for each pixel category from each section (10 teeth reflecting 5 sections per animal) were recorded and summed. Summation of mean values as grouped by genotype was used for the final analysis.

To measure molar shape and size a surface rendering of these teeth (M1, M2 and M3, respectively) was generated (e.g., [18]–[20]). Measurements were made from the lingual and crown perspective. For these measures set-points that for establishing basic measures of the anterior to posterior and buccal to lingual dimensions between cusps were used to provide comparable markers of length and width for each molar. Distances between these markers provide overall means that when summed afford statistical comparisons (Student's T-test) between the summed means of individual α7^WT^ and α7^KO^ mice. For enamel measurements, the mandibular incisor enamel was distinguished and the volume compared with total tooth volume that was easily distinguished by pixel intensity of these respective structures. We greatly appreciate the help of Douglas Vetter (Tufts University) and Michael McIntosh (University of Utah) who shared with us the nAChRα9^KO^ mice for this study and to Marina Picciotto (Yale University) for providing preserved heads from nAChRβ2^KO^ mice.

## Results

### The Expression of α7 Changes During Tooth Development

**Figure 1:**
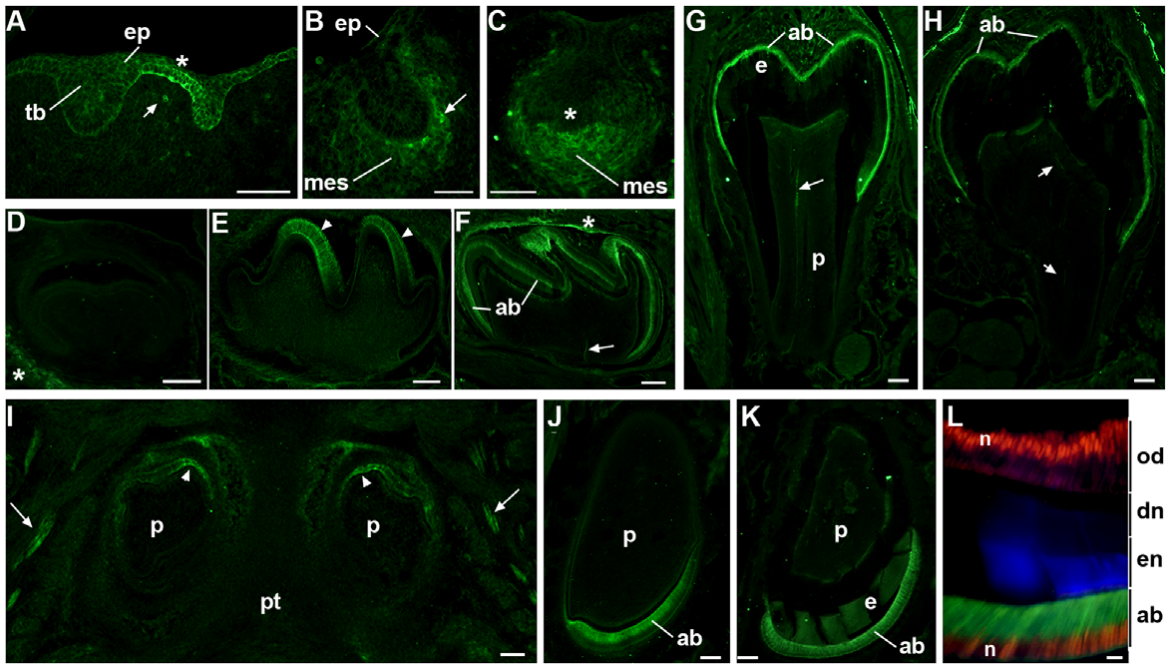
The expression of α7^GFP^ changes during different developmental stages of tooth development. A) Sagittal sections prepared of the E11.5 oral cavity shows α7^GFP^ expression, as detected by immunohistochemical staining for GFP expression, in the thickened dental lamina (asterisk) of the oral epithelium (ep) adjacent to or between developing tooth buds (tb). Only occasionally do cells of the underlying tissues including developing mesenchyme (mes) express detectable α7^GFP^ (arrow). B) E13.5 epithelial α7^GFP^ is no longer detected, but some cells in the developing mesenchyme (mes) at the border with the invaginated epithelium express α7^GFP^ (arrow head). C) E14.5 (bud stage) cellular expression of α7^GFP^ is enriched in cells of the condensing mesenchyme where it is particularly intense adjacent to overlaid epithelium and the primary enamel knot (asterisk). D) The expression of α7^GFP^ rapidly diminishes after E14.5 and is not detected in the E16.5 bell stage tooth organ except in surrounding neuronal processes (asterisk). E) α7^GFP^ expression returns in the E18.5 late bell stage where it is restricted to epithelial derived ameloblasts (arrow heads). F) P4 ameloblasts (ab) express α7^GFP^ and pioneering nerve fibers are also present (arrow) as well as in some residual nerve fibers still surrounding the tooth organ (asterisk). G) Molar teeth at P7 show α7^GFP^ expression by ameloblasts (ab) and the notable expansion of the enamel layer (e). A nerve fiber is also seen (arrow) in the dental pulp (p). H) By P12, ∼2days prior to eruption, the molar enamel is essentially complete and ameloblasts (ab) are degenerating and there is a coincident decrease in intensity of the α7^GFP^ signal. Again, nerves innervating the tooth are present arrow. I) In the incisors α7^GFP^ expression is initiated somewhat earlier than molars where it can be seen in this horizontal section of ameloblasts at the most dorsal-medial aspect of these developing teeth of the maxillary group (arrowheads). Trigeminal nerves (arrows) are also revealed by α7^GFP^ expression. The dental pulp (p) and pallet (pt) are identified. J) At birth (P0) there is strong expression of α7^GFP^ by all ameloblasts (ab) as seen in this mandibular incisor. The dental pulp is noted (p). K) By P12 α7^GFP^ expression is still observed in ameloblasts (ab) and the increase in enamel (en) deposition is apparent (enamel auto-fluorescence also appears green). M) Increased magnification of the P12 incisor stained for coincident expression of α7^GFP^ (Green) that is seen in the ameloblast cell bodies (ab) and the nuclear transcription factor, Cux1 (red) which identifies nuclei (n) in both ameloblasts (ab) and odontoblasts (od), which do not express α7^GFP^. The enamel (en) is auto-fluorescent (blue in this merged image) and the dentine (dn) layer is identified. Bars = 100 µm (A–J); 50 µm (L).

The temporal and spatial patterns of α7^GFP^ expression differ dramatically throughout tooth development (Fig. 1; [5], [6]). At the time of tooth involution of the dental epithelium (E11.5), α7^GFP^ is detected mostly in the intervening epithelial cells and it is absent in the regions of thickening leading to the dental lamina (Fig. 1A). The detection of α7^GFP^ is first observed in cells underlying the thickening dental lamina leading to molar teeth at approximately E12.5, but this becomes clearly evident at the E13.5 bud stage where accumulating cells of the dental mesenchyme are identified by α7^GFP^ expression (Fig. 1B). The molar epithelial bud undergoes patterned folding into a cap-like structure formed from the prominent epithelium as it invaginates to overlay the mesenchyme which is evident in the E14.5 cap stage (Fig. 1C). At this stage the α7^GFP^ labeling of cells in the condensing mesenchyme adjacent to epithelium underlying the primary enamel knot (PEK) is evident. Some weaker and inconsistent α7^GFP^ expression is detected in scattered cells that surround the PEK. The prominent expression of α7^GFP^ in E14.5 mesenchyme ends dramatically at E15.5 and it is effectively absent from all tooth organ structures by E16.5 (Fig. 1D).

The expression of α7^GFP^ reappears as the molar tooth enters the late bell stage (approximately E17.5 to E18.5; Fig. 1E). From this time forward molar ameloblasts express α7^GFP^ (Figs. 1F,G) until the enamel is deposited and the ameloblasts undergo degeneration (e.g., P12 in Fig. 1H) near the time of first mandibular molar eruption (∼P14; [21]).

The incisor teeth exhibit a similar pattern of α7^GFP^ expression. As shown in Figure 1I, α7^GFP^ expression is initiated by pre-ameloblasts of the maxillary incisors starting at E16.5. This begins in the medial aspect relative to the central axis and the expression expands rapidly to include all ameloblasts by E17.5 (not shown). Ameloblasts of the incisors express α7^GFP^ thereafter (as shown for P1 (Fig. 1J) and P12 when the enamel layer is expanded (Fig. 1K). Closer inspection of the P12 incisor ameloblast (Fig. 1L) reveals cytoplasmic GFP staining in the cytoplasm and the expected exclusion of tauGFP from the nucleus. This pattern of expression and the absence of α7^GFP^ in odontoblasts is seen when sections are co-stained for the nuclear maturation marker, Cux1 (Fig. 1L).

### α7^GFP^ Expression Distinguishes Tooth Innervation During Development

**Figure 2:**
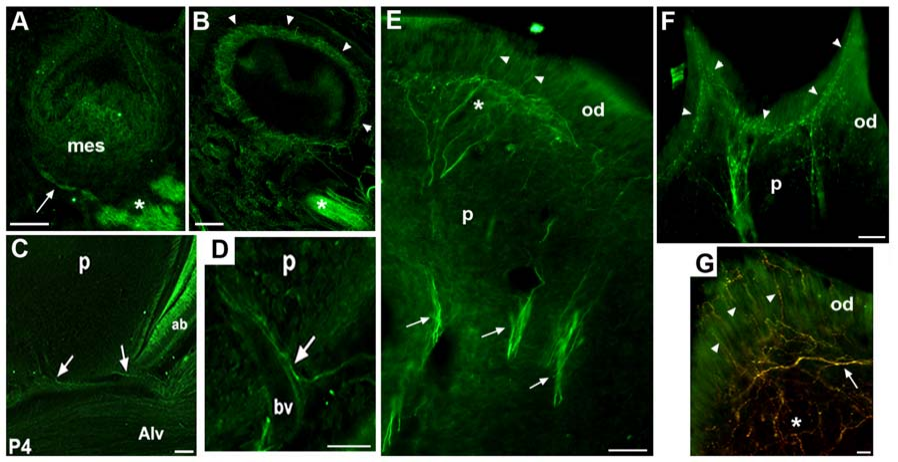
Expression of α7^GFP^ identifies all stages of tooth innervation. Neurons of the trigeminal system innervate the tooth and they also express α7^GFP^ which is visible in these nerves throughout tooth development by virtue of the tau-fusion component that directs the GFP to the axon. A) Pioneering processes of the molar nerves (arrow) express α7^GFP^ at E14.5. These extend from the trigeminal alveolar branches of both the maxillary and mandibular (shown, asterisk). Also seen is a single process that is present on the buccal side in the vicinity of the mesenchyme (mes) sheath. Extensions to the lingual aspect are present at E15.5 (not shown). B) By E16.5, when weak or essentially no expression of α7^GFP^ is detected in the structures of the developing tooth organ, the labeled molar nerve fibers (arrow heads) expand to populate the region surrounding the tooth organ to form a neuronal net. The mandibular alveolar nerve is identified (asterisk). C) Nerve fibers identified by α7^GFP^ do not enter the tooth until after birth. In our mice the loss of the ‘neuronal net’ surrounding the tooth and the entry of pioneering molar nerve projections (arrows) are well-defined in the P4 dental pulp (p). The alveolar nerve (Alv) and α7^GFP^ expressing ameloblasts (ab) are also seen. D) Increased magnification at the base of the P4 tooth shows the pioneering molar neuronal process (arrow) closely follows tracts established by previously formed blood vessels (bv) in the dental pulp (p). E) In the P12 molar the molar nerve fibers (arrows) have reached the odontoblast layer and formed a complex neuropil (asterisk) near the interface between the dental pulp (p) and the odontoblasts (od). Some fibers extend into the odontoblast cell layer (arrow heads). F) Increased magnification of the odontoblast layer of the P12 molar stained for α7^GFP^ shows the extensive neuronal patterning (arrowheads) under the odontoblast (od) cell layer. Also evident is the beaded structure of these neurons suggestive of sensory trigeminal neurons. Dental pulp (p). G) An image from a double labeled section (α7^GFP^ and the neuronal marker, TuJ1(red)) shows the neuronal network underlying the odontoblasts (od). Note the presence of TuJ1 labeled (red, asterisk) and double labeled neurons (yellow, arrow) that are usually more closely aligned to the odontoblast cell layer border. The double-labeled processes enter the odontoblast layer (arrowheads) and exhibit a beaded morphology consistent with sensory neuronal terminal. Bars = 100 µm, A,B; 50 µm, C–G.

The expression and function of α7 is commonly studied in terms of the nervous system or in peripheral systems where it also contributes to modulating the pro-inflammatory response [1]. While examining the tooth organ development, we also detected α7^GFP^ in nearby nerve fibers in patterns that closely coincide with previous studies of this process [22]–[24]. The detection of α7^GFP^ is seen first at E14.5 in the pioneering processes of the molar nerve (Fig. 2A) although occasional fibers at E13.5 are inconsistently detected (not shown). These processes originate as branches from their respective superior or inferior divisions of the trigeminal alveolar nerves (Fig. 2A) and they co-label with nerve growth factor p75 (not shown). Initially the molar nerves extend to the buccal side of the dental mesenchyme (Fig. 2A) but by E15.5 the first bifurcations to the lingual aspect are seen (not shown; [25]). At E16.5 when α7^GFP^ is essentially absent from the tooth organ (Fig. 1D), the prominent nerve net that surrounds developing molars is detected by α7^GFP^ immunostaining (Fig. 2B; [26]). The nerve plexus surrounding the tooth diminishes dramatically by birth, but α7^GFP^ prominently labels the pioneering fibers of the molar nerve that at P4 are seen to enter the tooth at the base of the molar dental papilla (Fig. 2C,D). These single nerve projections penetrate at sites coincident with the location of blood vessels (Fig. 2D; [12], [27], [28]). Thereafter, α7^GFP^ expression in molar nerves reveals a more extensive pattern as these processes are elaborated and become more widely distributed within the tooth pulp (e.g., P6; Fig. 2E). This extensive elaboration is particularly apparent in the P11–P12 molars (Fig. 2F,G) where an extensive array of molar nerve fibers is identified by α7^GFP^ that begin as aggregated tracts at the base of the dental papilla and then branch to form an extensive and dense fiber complex in the vicinity of the odontoblasts (Fig. 2F). Some of these fibers enter the odontoblast layer, although they do so sparingly (Fig. 2G; see [29]). The identity of α7^GFP^-labeled fibers and greater detail regarding their origin in the trigeminal ganglia is the topic of a future study. What is evident is that expression of α7^GFP^ by neurons that innervate the tooth is an excellent marker of this aspect of developmental patterning.

### Mesenchyme cells expressing α7^GFP^ have an instructive role in tooth patterning

**Figure 3:**
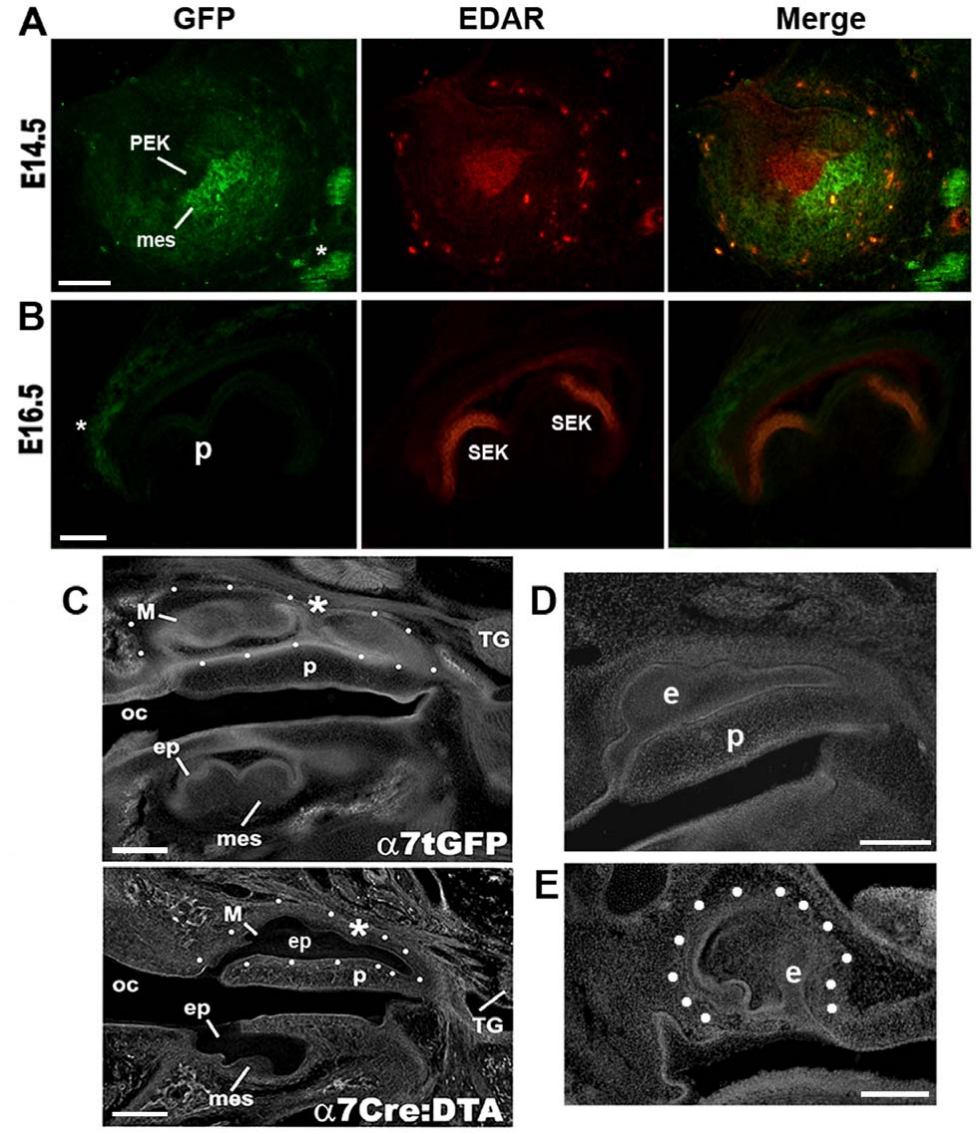
The expression of α7^GFP^ by cells in the mesenchyme and the impact of α7-cell lineage ablation on tooth organ ectodermal patterning. A) At E14.5 cells expressing α7^GFP^ are accumulated in the condensing mesenchyme (mes) adjacent to the epithelial primary enamel knot (PEK) which is identified by immunoreactivity to EDAR (red). The merged images reveal the close proximity between the EDAR stained PEK and the accumulated α7^GFP^ identified cells of the mesenchyme. Also present are some scattered cells in the dental papilla surrounding the mesenchyme that occasionally express α7^GFP^ which are also EDAR positive (arrow) and they co-label with the macrophage marker, IBA1 (not shown). Fibers of the trigeminal alveolar nerves are identified by α7^GFP^ labeling in the lower right (asterisk). B) In the E16.5 molar tooth EDAR identifies secondary enamel knots (SEK) that promote cusp formation, but no α7^GFP^ expression is detected except in surrounding nerve fibers (astrisk). Macrophages identified by IBA1 are present and even abundant at this stage, but they do not express either α7^GFP^ or EDAR after E15.5 (not shown). **C**) Conditional ablation of mesenchyme cells expressing α7^Cre^ using the Rosa26-LoxP(DTA) strategy described in the text and previously [16] results in abnormal tooth patterning. Shown are phase contrast images comparing sagittal cross sections taken at the same anatomical level from an E16.5 embryo of either the α7^GFP^ (control mouse; upper panel) or an α7^Cre∶DTA^ (lower panel). In these images the α7^GFP^ maxillary molar group (M) is outlined by white dots and the normal division between two of these molars is identified by an asterisk, but this separation is often absent in the α7^Cre∶DTA^ embryo (asterisk, lower panel). Also note the entirely different shape of mandibular molar in the lower panel. Particularly notable is the extensive overgrowth of the epithelium (ep) and decreased size of the mesenchyme (mes) components in the α7^Cre∶DTA^ embryo. The trigeminal ganglion (Tg), palette (p) and oral cavity (oc) are labeled. D–E) Additional examples of maxillary molars for litters of the α7^Cre∶DTA^ conditional ablation that were stained with nuclear dye 4′,6-diamidino-2-phenylindole (DAPI). In (D) this molar has an appearance similar to that of the corresponding molar in C (the same abbreviations are used). This overgrowth of the epithelium and the absence of three definable molars is common. E) This maxillary molar tooth from another α7^Cre∶DTA^ E16.5 embryo (outlined by dots) again illustrates the diversity that is possible in molar tooth shape in these E16.5 developing structures. Bars = 100 µm, (A,B); 200 µm, (C–E).

Because mesenchymal cells provide an instructive role in the signaling the PEK and controlling epithelial growth during tooth patterning, the accumulation of cells expressing α7^GFP^ in the condensing mesenchyme (Fig. 1C) suggested that α7 could contribute to modulating the interactions that control tooth size and shape [30]–[33]. One well-characterized marker of the PEK is the tumor necrosis factor superfamily member, ectodysplasin A receptor (EDAR). Abnormal function of this receptor contributes to phenotypes that include abnormal tooth size and/or shape [34]. To examine this, sections from embryos at E14.5 were co-stained for simultaneous detection of α7^GFP^ and EDAR expression (Fig. 3A). As expected EDAR immunostaining clearly identifies the PEK (E14.5, Fig. 3A) and the accumulation of α7^GFP^ labeled cells in the condensing mesenchyme immediately opposite the EDAR-identified PEK is evident. In addition to the accumulation of mesenchymal cells adjacent to the PEK as identified α7^GFP^, scattered cells in the dental papilla surrounding the mesenchyme also co-label for EDAR expression (Fig. 3A). The majority of these cells have a classic spider-like morphology characteristic of macrophages whose identity was confirmed based upon co-labeling with the markers IBA1 (not shown). The labeling of the PEK by EDAR was evident as early as E13.5 and it persisted into the E15.5 embryo. In contrast, α7^GFP^ expression was first seen in scattered mesenchymal cells at E13.5 (Fig. 1B), but it was essentially absent by E15.5 (not shown). Thus, at the E13.5–E14.5 developmental stage cells of the condensing mesenchyme express α7^GFP^ and accumulate adjacent to the PEK (identified by EDAR expression) and some scattered macrophages in the dental papilla express both α7^GFP^ and EDAR.

In the E16.5 molar EDAR is also expressed in the secondary enamel knots (SEK) that are important to shaping the molar cusps. However, α7^GFP^ is essentially absent from the tooth organ (Fig. 3B) and macrophages that are still present as revealed by strong IBA1 expression (not shown). Again, at this stage we observe only staining for α7^GFP^ in adjacent nerves (not shown, see Fig. 2). This argues that the expression of α7^GFP^ and EDAR are not interdependent?

### Ablation of the α7^Cre^ cell lineages, but not functional α7knock-out (α7^KO^), impacts upon tooth patterning

Since coordinated signaling between the epithelial∶mesenchyme at E13.5–E14.5 is crucial to the basic determination of tooth shape and size [5], [33], we examined if α7 expression directly impacts upon either parameter. To begin we first determined if the mesenchymal cells expressing α7^GFP^ participated in instructive signaling of the epithelium or if they are expendable to the early tooth determination. For these measurements we used a genetic approach to ablate the cells expressing α7 though examining the offspring of α7^Cre^×ROSA26-LoxP(diphtheria toxin A (DTA)) crosses and then examined these embryos at E16.5 [16]. In these embryos (termed α7^Cre∶DTA^) the cells expressing α7^Cre^ recombine the inactive DTA element whose subsequent production from the Rosa26 promoter results in the death of those cells thus providing a highly specific ablation of the mesenchymal cells that express α7^Cre^. As shown in Figure 3C, in α7^Cre∶DTA^ embryos, where α7^CRE^ expressing cells of the mesenchyme have been ablated, the overlying epithelium exhibits extensive overgrowth. The most common defect occurs in the maxillary molars where the epithelium extends across the pallet surface and, as anticipated, there is a substantially reduced mesenchyme (Figs. 3C–E). To date we have observed only one or occasionally two of these developing molar structures per quadrant suggesting that either a third molar does not form or possibly two or more of these fuse to generate the extended structure (not shown). Also evident is considerable variability in the phenotype between embryos, even those that are litter-mates. For example, while the maxillary molars in both Figure 3C (lower panel) and Figure 3D exhibit a similar morphology, others exhibit a dramatically altered morphology (Fig. 3E). This is consistent with our previous findings where other birth defects that result from the ablation of the α7^Cre∶DTA^ lineage, including spina bifida, also exhibit considerable variation in the severity of the defect between embryos [16]. Nevertheless the mere existence of these distorted molars is consistent with the results from the α7^GFP^ mouse where earlier stages of dental placode formation are independent of α7 receptor expression (Fig. 1). Thus, the ablation of α7^Cre∶DTA^ mesenchyme corresponds with the overgrowth of the overlying epithelium indicating the active and instructive role in ectoderm patterning by these cells.

**Figure 4:**
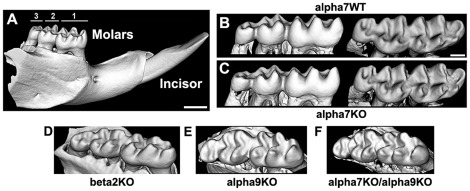
Functional ablation of α7 (α7^KO^) does not alter tooth shape or size. A) A typical high resolution surface rendered μCT scan of the α7^WT^ mandible (Methods). This illustrates the typical appearance of the molars (numbered) and an incisor. B–C) Examples of μCT rendered images of α7^WT^ and α7^KO^ (WT or KO, respectively) mandibular molars (lingual aspect) or the same teeth rotated to reveal the crown and cusps. As shown in these images, overall molar size and morphology did not differ between these respective genotypes. D–F) CT-images are shown of the mandibular molars from a nAChRμ2^KO^ (D), nAChRμ9^KO^ (E) or (F) the double knock-out (α7^KO^/α9^KO^). The similarity of size and shape among teeth of these various genotypes indicates that under normal physiological conditions of development the absence of α7 function does not impact upon this developmental parameter. Bars = 300 µm (A); 100 µm, (B–F).

We next asked if the absence of α7, as in the α7 knock-out (α7^KO^) corresponds with any defects in tooth patterning such as in tooth size or shape. For this analysis we imaged and then compared the molar teeth of both α7 wild-type (α7^WT^) and α7^KO^ mice using high resolution small animal micro-computed tomography (μCT) scanning (Fig. 4). Renderings of the 3-D surface image for a total of 9 α7^WT^ and 10 α7^KO^ adult mice (3–4 months old) were then generated and measured for the width, length and inter cup distances among the 3 molars (Fig. 4A and Methods). As shown for a typical mandibular molar set (Fig. 4B,C), the shape and size of molars were indistinguishable between α7^WT^ and α7^KO^ mice. One possibility to explain this result would be that another nAChR compensates for the absence of α7 in the α7^KO^, thus obscuring a direct effect by α7 function [1]. To test this possibility, we obtained the α9^KO^ mouse and heads from the β2^KO^ mice (Methods). The α9 receptor is also a homomeric nAChR and shares several key properties with α7 that might suggest it as a candidate for compensation [1]. The β2 subunit is broadly expressed in nAChRs related to the addictive effects of nicotine and whose absence is related to multiple phenotypes that reflect the function of these receptor subtypes [1], [35]. Examination of the teeth from both the α9^KO^ and β2^KO^ mice (Figs. 4D,E) revealed no differences from the teeth of these mice and those of wild-type mice in terms of overall shape or size (Fig. 4). The availability of the α9^KO^ mice offered us the opportunity to generate the double α7^KO^/α9^KO^ mouse (Methods) and directly examine the issue of possible compensation by these two receptors. In all cases, mice from the double subunit knock-out, α7^KO^/α9^KO^, also did differ from the wild-type controls (Fig. 4F). Thus, under normal physiological conditions of embryonic development, α7 appears to be expendable and its absence is not compensated for by α7.

### Functional knock-out of α7 corresponds with altered tooth enamel structure

**Figure 5:**
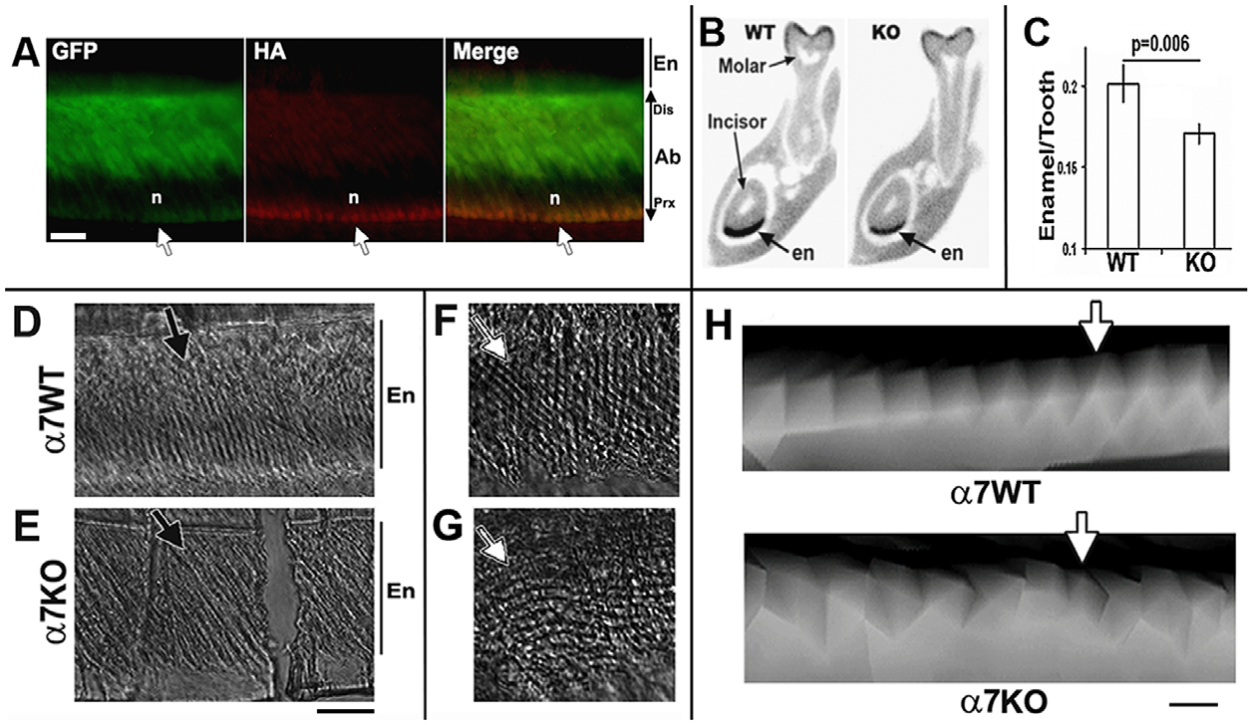
Ameloblasts express α7^GFP^ and enamel structure is altered in α7^KO^ mice. A) Image of mandibular incisor (P12) showing co-labeling for GFP (α7^GFP^) and the α7-associated hemagglutinin epitope tag (HA). GFP fills the cells expressing α7 (except the nucleus which excludes this protein), the HA immunostaining is located only on the cell's basal (proximal) surface (arrow) indicating the majority of α7 receptors are accumulated at this site. The nuclear layer (not stained) is identified (n). The labels identify the ameloblast (Ab) and enamel (en). Distal (dis) and proximal (Prx) directions are noted. B) A virtual coronal cross-section made through the same region of an adult mouse of the α7^WT^ or α7^KO^ using high resolution μCT scanning (see methods). Because incisors are continually renewed, the enamel density is very consistent among animals whereas similar calculations of molar enamel volume were not measured due to the variability among animals presumably due to enamel attrition. This can be seen along the molar crown where the intensity of black varies and is not always continuous. The incisor and first mandibular molar are identified. In these images surrounding bone and material of similar or less density is colored grey whereas enamel (en) is in black. The volume of the mandibular incisor enamel and the total cross-sectional volume was calculated and summarized from images similar to these of multiple animals (see text). Note that the α7^KO^ enamel appears to be ‘thinner’ relative to the matched image taken from an α7^WT^ litter mate. C) Results of quantitation of enamel volume from 5 bilateral μCT image slices at matched anatomical intervals (dorsal to posterior) between α7^WT^ and α7^KO^ mice. The data from each section were averaged to obtain an animal mean that were then summarized for all animals of each genotype to generate the graph shown. The relative volume of enamel is consistently ∼25% less in the α7^KO^ than in the α7^WT^ (P = 0.006). Error bars are +/− standard error of the mean. D–E) Phase contrast microscopy of enamel from the mandibular incisor of an adult α7^WT^ (WT) or α7^KO^ (KO) mouse incisor. The enamel rod layer is visible (black bars identified by En) as parallel arrays in roughly perpendicular columns (arrows) relative to the external surface (bottom of photo). F–G) A view at greater magnification of a cross section shows the regularity of the rod lattice of the α7^WT^ enamel that appear as less regular and often tilted relative to the perpendicular axis in the α7^KO^ (arrows). H) High resolution μCT images of the lower mandibular incisor near the site of eruption showing enamel rods (collections of bundled prisms). The columns within the enamel in the α7^WT^ (G) are regular and neatly ordered (arrow). This is in contrast to the α7^KO^ (H) where columns are irregular and often are difficult to resolve. Although not apparent in these images, the spacing between the columns is not different indicating that the overall size and growth rate does not differ between mice of these genotypes (not shown). Bars = 50 µm (A); 100 µm, (D–G); 20 µm (A).

As shown in Figure 1, after E17.5 there is a recurrence of α7^GFP^ expression in the developing teeth that is limited to pre-ameloblasts and mature ameloblasts, the cells that produce the highly mineralized hard enamel surfaces. However, because GFP is a marker of receptor transcription we determined if the α7 receptor protein is also being expressed by ameloblasts. The detection of α7 is often complicated by the inconsistency in the specificity and sensitivity provided by commercially available antibodies to this receptor [36]. To overcome this we inserted an HA-epitope tag into the α7 C-terminus for detection of α7 using the anti-HA antibodies (Methods and [16], [17]). Sagittal sections of the α7^GFP^ P10 mandibular incisor were prepared and co-labeled for the detection of both GFP and anti-HA expression (Fig. 5A). The GFP labeling fills essentially the entire cell, except for the nucleus (Fig. 5A and Fig. 1L). This is in contrast to the detection of HA epitope expression, which locates the α7 receptor protein to the proximal (basal) side of the ameloblast (Fig. 5A). This suggests α7 is highly localized to the ameloblast basal membrane at or near site of the concentration of metabolic mechanisms related to calcium acquisition and regulation during enamel formation [37], [38].

To examine if α7 alters ameloblast function, we used the same animals as above to examine the enamel structure as revealed by μCT analysis. Of note is that the mouse model offers a particular advantage for these types of analyses because we can focus on incisors which erupt throughout the life of the animal. This makes the mouse incisor an ongoing model of tooth morphogenesis including the constant regeneration and maturation of ameloblasts and the formation of new enamel to the ventral aspect of this tooth [38], [39]. These measurements took advantage of the high mineralization density of mature enamel as a simple way to visualize enamel volume and structure in images collected through μCT (Fig. 5B, [20]). We first noted that the enamel density of the α7^KO^ mandibular incisor appeared to be ‘thinner’ than comparable virtual sections taken from in α7^WT^ (Fig. 5b). To quantitate this apparent difference in multiple animals, we used a stereological approach. Virtual serial μCT sections at 500 µm intervals of the mandibular incisors (5 per tooth for each tooth and each animal) at comparable anatomical levels near the site of eruption from both α7^WT^ and α7^KO^ were prepared, partitioned into enamel versus total cross-section density, and the ratio between these respective areas calculated (Fig. 5C; Methods). We did not examine molars quantitatively because there was inconsistency in enamel density on the apical tooth surface presumably due to uneven attrition. The results of these analyses revealed the α7^KO^ enamel volume was on average reduced by 20 percent compared with α7^WT^ litter mates ((p>0.006; Fig. 5C). Also, because sufficient material was available, we did the same analysis of the α9^KO^. In these animals incisor enamel volumes were similar to controls (not shown) indicating further that the differences measured are specific to the α7 subunit.

In addition high resolution images there was also noted an appearance of altered organization of the enamel rods (aggregates of crystalline-like prisms) in the α7^KO^ relative to the α7^WT^. These differences are visible in phase microscopy images of sections from the mandibular incisors to these respective α7 genotypes (Fig. 5D,G). As seen is these images of the mandibular incisor, the enamel rods of the α7^KO^ appear less uniform and exhibit a greater slant relative to the vertical axis when compared to similarly oriented preparations of the α7^WT^ (Fig. 5D,E). Also, at greater magnification the α7^KO^ enamel appearance is more striated and at greater magnification suggests that the crystalline pattern has become misshapen when compared to the regular rhombomeres of the α7^WT^ (Fig. 5F,G).

High resolution CT scans of the mandibular incisor also confirm this enamel irregularity (Fig. 5H). Here the enamel shape of the bundled prisms of the α7^KO^ is distorted relative to the control. Quantitation of these images has been limited in part due to variability in the ability to image α7^KO^ enamel at high resolution in part because it is more difficult to separate it from surrounding tissues relative to the α7^WT^ for image rendering visualization. Also, the measurements of the inter-array distances revealed no difference in this parameter between these mouse genotypes (not shown). This suggests that the enamel alteration in the α7^KO^ likely reflects a change in enamel composition, although this hypothesis remains to be examined.

## Discussion

In this study we describe the expression of α7 during development of the teeth and show that for this nicotinic receptor the distinct spatial and temporal differences in its expression suggests functional pleiotropy in the tooth developmental process. Notable is the initial expression of α7^GFP^ in the E13.5 and E14.5 mesenchymal cells during endoderm determination and later (>E17.5) by differentiating epithelial derived ameloblasts. Throughout tooth development α7^GFP^ expression also serves to identify tooth innervation and maturation. We have presented evidence to suggest that α7 participates directly in the outcome of tooth development. The most direct is the alteration of mandibular enamel structure present in the α7^KO^ mouse. Speculations on how α7 could contribute to ameloblast-directed enamel formation function include mechanisms that are uniquely related to this nAChR. One could be related to the receptor's high permeability to calcium [1] where it would participate directly in ameloblast sequestration of calcium. A second possibility is that α7, as is seen in other systems [1], [2], contributes to the normal stasis of metabolic or cell signaling processes that in this case would be related to enamel formation. For example, the impact by α7 may be on regulating secreted matrix-metalloproteinase [40], [41] and that similar mechanisms used by ameloblasts to define inter-crystal adherence and subsequent packing of enamel prisms into enamel bundles could be altered [42]–[45]. Finally, α7 activity is well characterized to modify several transcriptional systems including some directly related to normal developmental and differentiation processes such as CREB, NF-kappaB and p38MAPK [46]–[51]. Consequently, the direct modulation of intracellular signaling of transcription by α7 could impact upon multiple pathways leading to an imbalance that would compromise normal ameloblast function including issues pertaining to maturation and cell longevity.

Another component of α7 participation in tooth formation occurs in the initiating mesenchyme during ectodermal organ determination. Although no impact on tooth shape or size was found in the α7^KO^, ablation of cells expressing α7 dramatically altered tooth development in the embryo consistent with the mesenchyme expressing α7 having an instructive role in this process. Several possibilities could explain this result. First, a well know aspect of development is redundancy in genes that perform critical roles. This is particularly evident during ectoderm patterning for genes where functional redundancy is common and lethality may not be observed unless there is dominant mutation or multiple activities are ablated simultaneously (e.g., see [52]). Even for nAChRs individual knock-out of the β2 or β4 subunit is well tolerated, but the dual knock-out exhibits severe consequences including autonomic dysfunction [53]. While we did examine the possibility of additional nAChR subunit contribution, this study is incomplete given the number nAChRs and the possibility of many different receptors that could be expressed [1]. More likely is that if α7 does contribute to mesenchyme function, this may only be revealed only if the system is perturbed. There is precedence for this possibility since the regulatory importance of α7 towards modulating the pro-inflammatory response is observed during an inflammatory event and is not readily detected in animals (including the α7^KO^) under normal physiological conditions [1], [2].

In the context of this discussion, nicotine and nicotinic receptors are most commonly associated with the use of tobacco products. Certainly considerable epidemiological evidence supports a negative influence by nicotine in both development and even later in life oral health (both in development and after birth), but the mechanisms explaining the diversity of effects and varied susceptibilities of individuals to challenge by this exogenous remain ill-defined. We have not examined the impact of α7 functional modulation on tooth development. One obvious way to do this is through administration of the α7 agonist, nicotine, which has been related to slowed tooth development, reduce the breadth of the first molar in rodents and produce dental papilla necrosis in culture [54]–[56]. As noted, the impaired secretion of the enamel matrix has also been reported in animals exposed to pre- or post-natal nicotine, a process that in humans may not be relevant until post-natal periods when enamel deposition completes the permanent teeth just prior to their eruption. All of these potentially fit with the observations defined in this study as to how α7 functional pleiotropy expression could influence tooth development depending upon the timing and duration of the event that modifies the function of this receptor.

Finally, a more speculative line of investigation may involve relevance to certain psychiatric disorders where deficiencies in α7 expression and function are reported to contribute to the onset and disease severity [57], [58]. The occurrence of tooth abnormalities in these disorders within the context of this study becomes of potential relevance [59]. Thus, an intriguing avenue of study is the possibility that both genetic and epigenetic factors could contribute though altering α7 function to create a spectrum of phenotypes whose variability, combinational complexity and severity will directly depend on the nature, amplitude and timing of the respective insult(s).
